# Emerging Therapeutic Role of PPAR–α in Cognition and Emotions

**DOI:** 10.3389/fphar.2018.00998

**Published:** 2018-10-02

**Authors:** Khalin E. Nisbett, Graziano Pinna

**Affiliations:** The Psychiatric Institute, Department of Psychiatry, University of Illinois at Chicago, Chicago, IL, United States

**Keywords:** allopregnanolone, *Biomarker axis*, N-palmitoylethanolamine, PPAR–α, PPAR–γ, 5α-reductase, contextual fear responses, PTSD

Neurosteroids and the endocannabinoid system are increasing in relevance as themes in the studies of many disorders and diseases (Berardi et al., 2016; Basavarajappa et al., 2017; Rasmusson et al., 2017). Correspondingly, psychiatric disorders, like post-traumatic stress disorder (PTSD), correlate with changes in endogenous neurosteroid and endocannabinoid availability, which may be related to the cause of its comorbidity with general cognitive decline (Qureshi et al., 2011; Schuitevoerder et al., 2013), neuroinflammation (Jeon and Kim, 2016; Mendoza et al., 2016), and neurodegenerative disorders (Cummings, 1992; Chi et al., 2014). These neuropathologies also reduce the quality of life and increase the socioeconomic burden.

Furthermore, increasing evidence shows an association of chronic maladaptive brain changes with neuroinflammation in PTSD (Jones and Thomsen, 2013). It is marked by the upregulation of pro-inflammatory cytokines such as IL−1β, IL−6, IL−10, and TNF–α in the CNS (Minami et al., 1991; Cunningham et al., 1992; Mogi et al., 1994, 1996). Particularly, IL−1β plays a critical role in the activation of the HPA axis (Shintani et al., 1995), and in the hippocampal formation where it regulates stress-enhanced fear learning (Jones et al., 2015). As such, the association between neurosteroids and neuroinflammation is unsurprising (Purdy et al., 1991; Webster et al., 2015; Villa et al., 2016). Indeed, neuroactive steroids, allopregnanolone and deoxycorticosterone have also been shown to increase during acute stress to levels that activate the GABA_A_ receptor, and thereby influence the behavioral responses (Purdy et al., 1991). The chronic stress response has also been found to coincide with decreased cognitive function, especially learning and memory deficits (McEwen and Sapolsky, 1995), in episodic memory (Payne et al., 2006), and spatial learning and memory (Conrad, 2010). Chronic stress also negatively alters sleep patterns, social behavior, mood (Opp et al., 1988; Pinna et al., 2003, 2008; Hall et al., 2015; Olini et al., 2017 reviewed in Locci and Pinna, 2017), as well as decreases neurosteroids (Pinna et al., 2006, 2009; Bortolato et al., 2011), which affect synaptic plasticity (Serra et al., 2008; Fester and Rune, 2015) and neurogenesis (Wang, 2014).

The high prevalence of PTSD in the US makes it a high priority research topic. Approximately 7–12% of US adults are affected by mood and anxiety related disorders (Anxiety Depression Association of America, 2010-2016), while 4% of US adults (Harvard Medical School, 2007) and 20–30% of US veterans are affected by PTSD specifically (US Department of Veterans Affairs, 2015a). There are currently no reliable mechanisms or biomarkers to predict the onset or progression of PTSD, nor are there treatments that can consistently reduce the symptoms of PTSD. Currently, the only approved pharmacotherapies for PTSD are the selective serotonin reuptake inhibitors (SSRIs), however, these treatments have low response rates and only treat a small subset of patients (Hertzberg et al., 2000). The neurosteroid system is emerging as novel neuronal substrates in the pathogenesis of PTSD and its regulation may facilitate recovery (Yu et al., 2011; Zanettini et al., 2011; Litvin et al., 2013; Locci and Pinna, 2017; Pineles et al., 2018).

The goal of this opinion article is to examine the relationship between the endogenous fatty acid amides, including palmitoylethanolamide (PEA) and the biosynthesis of neurosteroids, particularly allopregnanolone, and their role in emotional and cognitive dysfunction in PTSD. Specifically, we focus on the function of the peroxisome proliferator–activated receptor (PPAR)–α, a target for PEA, which is best known for its role in reducing inflammation by decreasing cytokines, pro-inflammatory enzymes and oxidative stress. For this, PPAR–α agonists act as neuroprotectants in various neurological disorders like Alzheimer's disease, Parkinson's disease, multiple sclerosis, and cerebral ischemia (Zolezzi et al., 2017). However, recent literature in the field suggest that PPAR–α has emerged as a new target that is useful as a novel approach to treat mood disorders by engaging neurosteroid biosynthesis.

## The endocannabinoid system and the role of PPARs in cognition and emotions

The endocannabinoid system was curiously discovered in the 1990s because of the psychotropic effects that resulted from the use of *cannabis sativa* in medicinal and recreational settings (Di Marzo et al., 2004). The goal of early research was to elucidate the active agents, but, with time, research turned from the study of the psychotropic effects of the endocannabinoid system to the study of its medicinal properties. Eventually, treatment-oriented research revealed that the endocannabinoid system plays an important physiological role in homeostasis, pathogenesis and recovery in healthy and ill brain states (De Petrocellis et al., 2004), and is heavily involved in the regulation of emotions, cognition and stress (Viveros et al., 2005; Zanettini et al., 2011). The typical target of endocannabinoids in the CNS is the G-protein coupled, type-1, cannabinoid receptor (CB−1). Its role in pathogenesis and recovery is well investigated (Manzanares et al., 2004). However, much more recently, PPARs have emerged as new targets for cannabinoids and fatty acid amides for the regulation of pathophysiological functions, including inflammation, oxidative stress, alcohol addiction, and behavioral deficits (Le Foll et al., 2013; Mandrekar-Colucci et al., 2013; Rolland et al., 2013; Locci and Pinna, 2017; Rivera-Meza et al., 2017).

The PPAR family is a ligand-dependent, nuclear hormone receptor, transcription factor family of three isotypes: PPAR–α, PPAR–β/δ, and PPAR–γ (Fidaleo et al., 2014). Of the family, PPAR–β/δ is the least understood, yet it is known to have a role in the development of the CNS and cell survival (Berger and Moller, 2002; Abbott, 2009). PPAR–α and PPAR–γ have similar neurophysiological functions that include regulation of the redox response, neuroinflammation, neurogenesis, cellular differentiation, as well as secondary functions in the regulation of cognition, anxiety, and emotional behavior (Bordet et al., 2006; Bright et al., 2008; Panlilio et al., 2012; Fidaleo et al., 2014). PPAR–α and PPAR–γ are localized in brain regions that are selectively involved in the regulation of emotions and the stress response (Moreno et al., 2004). PPAR–α is most highly expressed in the basal ganglia, amygdala, prefrontal cortex and thalamic nuclei of healthy adults, with lower levels in the hippocampal formation (Warden et al., 2016). PPAR–γ is also highly expressed in the basal ganglia and amygdala, with lower levels in the hippocampal formation, and the thalamic nuclei (Moreno et al., 2004). The significance in the relationship between these regions and emotions has been extensively studied (Shin et al., 2006; Shin and Liberzon, 2010). Together, the basal ganglia, prefrontal cortex, amygdala, thalamus and hippocampus are all key components of the neuronal circuit for fear and anxiety (Shin and Liberzon, 2010), while the basal ganglia, prefrontal cortex and thalamus are critical to mediation of emotional drive and the planning of goal-directed behaviors—which are either exaggerated or depressed during a threat (Haber and Calzavara, 2009). The amygdala is crucial to learning threat-stimuli relationships and the expression of cue-specific fear (Davis, 1992). Its activity is heightened in PTSD, social phobias and related disorders (Shin and Liberzon, 2010). This hyperresponsivity of the amygdala likely dampens the responsivity of the prefrontal cortex, which manifests as hyporesponsivity in PTSD patients (Garcia et al., 1999; Shin and Liberzon, 2010). Additionally, the hippocampus which plays a fundamental role in memory acquisition, consolidation and retrieval, is likely influenced by the amygdala, especially in relation to threatening contexts (McGaugh, 2004).

PPAR–α activation has been shown as a natural response to stress, having the ability to mediate and modulate the stress response (Hillard, 2018). In healthy adults, PEA, an endogenous PPAR–α agonist, significantly increase after clinical stress tests, corresponding with increased cortisol levels (Dlugos et al., 2012). PEA levels increase when healthy individuals experience pain or a depressed mood transiently (Darmani et al., 2005). However, the levels of PEA in PTSD are low (Wilker et al., 2016), suggesting a significant role in emotion regulation. As such, endogenous and synthetic PPAR–α ligands have predictably and successfully stabilized emotions in preclinical models (Locci et al., 2017).

Enhanced fear memory, depressive-like behavior, and aggressive behavior are common characteristics of chronically stressed animals in murine models of PTSD that resemble human symptomology. PPAR–α activation has been assessed regarding its effect on this behavior. PPAR–α agonism rescued rodent behavior in response to stress induced fear. When PPAR–α was activated by exogenous PEA in socially isolated mice, a mouse model of PTSD, fear memory acquisition was reduced, and impaired fear extinction was rescued (Locci and Pinna, 2017; Locci et al., 2017). Similarly, PEA induced a dose-dependent anti-depressant effect (Yu et al., 2011), and reduced aggressive behavior that was blocked by pretreatment with antagonists (Locci et al., 2017). The relationship between PPAR activation and emotional regulation is further supported by its activity in neuroinflammation (O'Leary, 1990; Racke and Drew, 2008; Rolland et al., 2013; Esmaeili et al., 2015; Jeon and Kim, 2016), but even more so, by the localization of PPAR–α in brain areas that regulate mood and emotions.

In an analogous manner, the downregulation of PPAR–γ has been reported to exaggerate basal anxiety, enhance stress sensitivity and produce substantially different stress-induced neuronal activity in the amygdala and hippocampus (Domi et al., 2016). PPAR–γ antagonist, GW9662, produced an anxiogenic-like response, while PPAR–γ agonists did not affect basal anxiety-like behavior (Rosa et al., 2008). Similarly, treatment of rats with the PPAR–γ agonist, rosiglitazone, reduced the systemic response to acute stress, and reduced the heart rate in response to an acute restraint stress (Ryan et al., 2012). In this study, treated rats also showed a blunted hormonal response (corticosterone levels). However, in contrast to the above, young, unstressed rats treated with rosiglitazone showed an improved response in the hippocampal-dependent fear conditioning task in comparison to control rats (Gemma et al., 2004). This may point to an analogous role for PPAR–γ activation in the treatment of anxiety and/or depression.

## Role of allopregnanolone in cognition and emotions

3α,5α-tetrahydroprogesterone, also known as allopregnanolone, is a neurosteroid that can be synthesized *de novo* from cholesterol, or from its precursors, pregnenolone and progesterone (Pinna et al., 2006; Schüle et al., 2014). In the CNS, allopregnanolone can function to rapidly alter neuronal excitability by acting as a potent and positive allosteric modulator at post- and extra-synaptic GABA_A_ receptors, which are highly abundant in glutamatergic neurons (Pinna et al., 2000). These neurons participate in the circuit of fear, and are therefore involved in emotion and anxiety regulation (Möhler, 2012). As such, an imbalance of GABAergic neurotransmission, or endogenous neuromodulators results in abnormal regulation of emotion and abnormal stress responses (Möhler, 2012; Locci and Pinna, 2017). This inhibitory deficit is a known hallmark in anxiety and emotional disorders. Given that allopregnanolone directly binds this receptor, a reduction of allopregnanolone levels correlate to reduced GABA_A_ receptor activity and dysfunctional behavior (Pinna et al., 2008, 2009).

Intriguingly, the allopregnanolone level in the blood and CSF are reduced in patients of MDD, impulsive aggression, premenstrual dysphoric disorder, PTSD and other disorders of mood and emotions (Rasmusson et al., 2006, 2016; Schüle et al., 2014; Šrámková et al., 2017; Pineles et al., 2018; Rasmusson and Pineles, 2018). Another interesting phenomenon is the observation that females are twice as likely to experience PTSD as males; 10% of women who experience trauma develop PTSD, compared to only 4% of men (US Department of Veteran Affairs, 2015b). The gender difference in PTSD patients further indicates that neurosteroids may play a large role in the progression and recovery of these disorders, as the difference in neurosteroid concentration contribute to the biological distinction of the sexes (Mendoza et al., 2016). As a specific example, the allopregnanolone level in the CSF of female PTSD patients were 40% lower than in controls, and the allopregnanolone/dehydroepiandrosterone (DHEA) ratio negatively correlates with PTSD re-experiencing (Rasmusson et al., 2006). To this end, studies are being pursued to verify lower levels of allopregnanolone during pregnancy as a predictor of postpartum depression (PPD) (Osborne et al., 2016; Kanes S. et al., 2017).

Early studies have shown that allopregnanolone levels in the brain increase to levels that can activate the GABA receptors, during acute stressful events (Purdy et al., 1991). Subsequently, it has been further hypothesized that the enhancement of GABAergic transmission decreases HPA activity and contributes to the behavioral stress response (Cullinan et al., 2008). Protracted stress, on the other hand, downregulates allopregnanolone biosynthesis (Pinna et al., 2003; Matsumoto et al., 2005, 2007). Indeed, preclinical studies demonstrate that socially isolated mice, known to exhibit enhanced contextual fear responses and impaired fear extinction, also exhibit time-related decreases in allopregnanolone levels in neurons of the medial prefrontal cortex, hippocampus and basolateral amygdala (Agís-Balboa et al., 2006, 2007; Pibiri et al., 2008). The decrease of allopregnanolone was the result of reduced levels of 5α-reductase type I mRNA and protein following social isolation (Dong et al., 2001; Matsumoto et al., 2005, 2007). Hence, these findings suggest that allopregnanolone, its precursors, and analogs of allopregnanolone are suitable treatments for emotional regulation (Pinna and Rasmusson, 2014; Locci et al., 2017). For example, exogenous allopregnanolone attenuated the contextual fear response in a dose-dependent manner. In a similar murine social isolation model of PTSD, researchers showed that allopregnanolone treatment normalized HPA responsiveness and interrupted depressive- and anxiety-like behavior, which are hallmarks of clinical PTSD (Evans et al., 2012). Allopregnanolone analogs, BR351 and BR297, effectively decreased aggression in socially isolated mice, with a lower non-response rate than SSRI-treated mice (Locci et al., 2017). Given preclinical successes, allopregnanolone, its precursors and its analogs are currently being sort after and tested as treatments in psychiatric and related disorders. Recently, allopregnanolone (brexanolone) was evaluated in phase 3 clinical trials for its efficacy against PPD, and successfully achieved primary endpoint (Kanes S. J. et al., 2017). For the phase 2 clinical trial, women were given an intravenous infusion of allopregnanolone, and outcomes were measured using HAM–D (Kanes S. et al., 2017). Of 21 enrolled patients, 70% of treated vs. only 9% of placebo-receiving patients exhibited remission of depressive symptoms. Researchers hypothesize that the action of this drug includes the potentiation of GABA_A_ receptors (Kose and Cetin, 2017).

## The bridge between PPAR–α stimulation and allopregnanolone biosynthesis

The summaries above suggest that the role of allopregnanolone in the progression and recovery of psychiatric disorders is similar to the emerging role of PPAR–α. Importantly, these similarities are not limited to their function in emotion regulation. Comparable actions of PPAR–α and allopregnanolone have also been observed across cognition (Cuzzocrea et al., 2013; Fidaleo et al., 2014; Greene-Schloesser et al., 2014), neurogenesis (Ramanan et al., 2009; Fidaleo et al., 2014), neuroinflammation (Daynes and Jones, 2002), neurodegeneration (Naylor et al., 2010; Esmaeili et al., 2015), and substance use disorder (Le Foll et al., 2013; Blednov et al., 2015; Rivera-Meza et al., 2017). Raso et al. suggest that the PPAR–α and allopregnanolone are different substrates of the same mechanism, whereby PEA-induced activation of PPAR–α regulates the biogenesis of allopregnanolone in astrocytes (Raso et al., 2011). To this end, when astrocytes were treated with PEA *in vitro*, an increased expression of enzymes that are crucial to allopregnanolone biosynthesis [steroidogenic acute regulatory protein (StAR) and cholesterol side-chain cleavage enzyme (P450scc)] were reported along with increased cytoplasmic concentrations of allopregnanolone. This interdependent relationship between PPAR–α and allopregnanolone has also been alluded to in studies of pain perception. In studies of acute and persistent pain, researchers showed that the usual anti-nociceptive activity of PEA was reduced when activity of enzyme 5α-reductase and P450scc were blocked (Sasso et al., 2012). PEA restored enzyme expression and increased allopregnanolone level in the spinal cord. Further support for this relationship was shown when PEA was used as neuroprotector and regulator of the pentobarbital-evoked hypnotic effect (Sasso et al., 2010). In this case, PEA increased the expression of relevant enzymes and allopregnanolone concentrations in the spinal cord.

**Figure 1:**
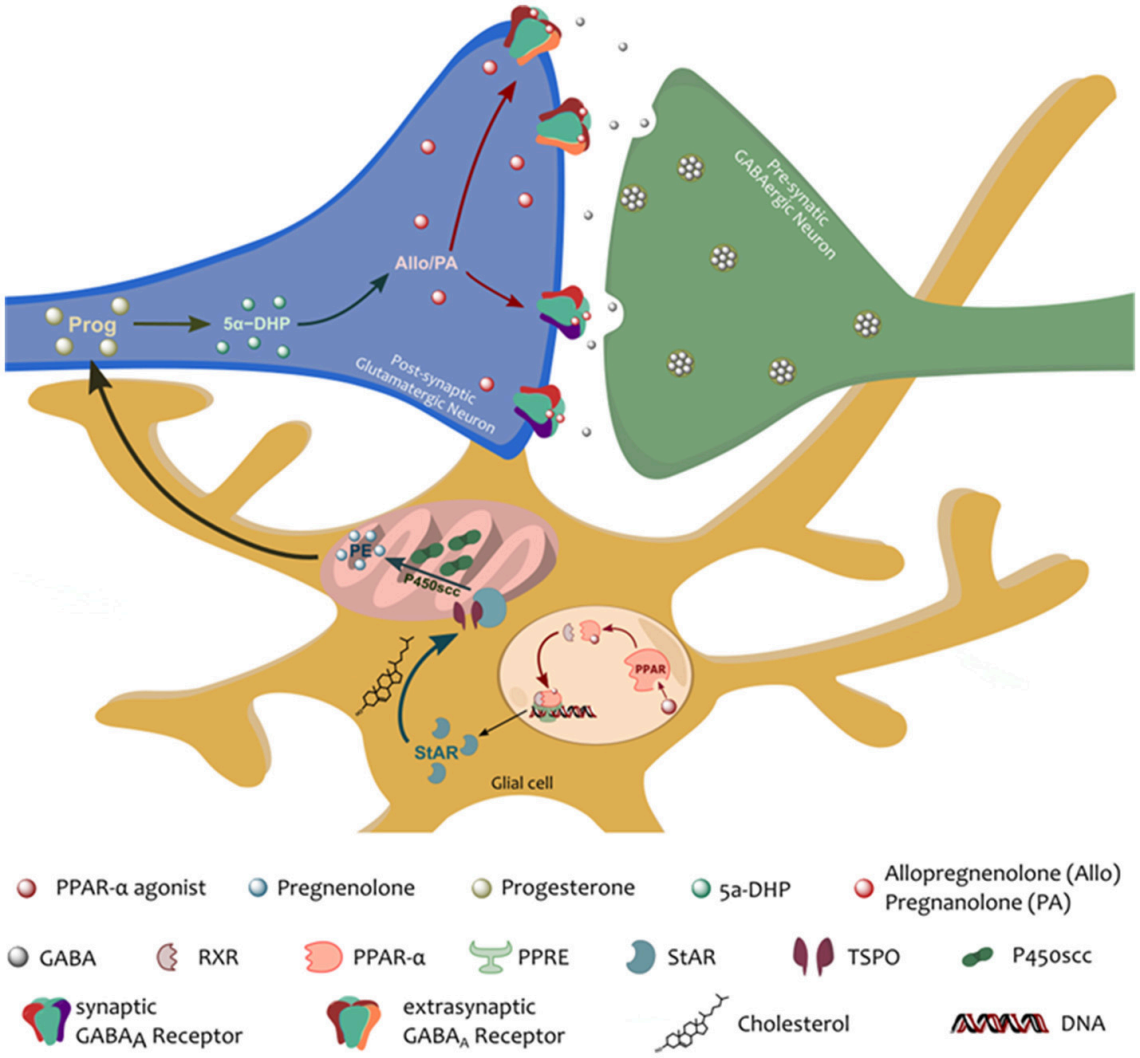
Schematic representation of the proposed *PPAR–*α*-allopregnanolone biomarker axis*. PPAR–α, following its activation by an endogenous (e.g., PEA) or a synthetic agonist, heterodimerizes with a PPAR–α-specific retinoid X receptor (RXR). The PPAR-RXR dimer then binds the PPAR response element (PPRE) in specific promoter regions that up- or down-regulate gene expression. PPAR–α activation would therefore normalize the stress-induced downregulation of neurosteroidogenic proteins, StAR and p450scc. StAR, is crucial to the translocation of cholesterol into the inner mitochondrial membrane. There, cholesterol is metabolized by the action of the P450scc into pregnenolone (the precursors of all neurosteroids). Pregnenolone can be further converted to progesterone and 5α-dihydroprogesterone (5α-DHP) by the action of 5α-reductase type I. 5α-DHP can then be converted by 3α-hydroxysteroid dehydrogenase into allopregnanolone (Allo) and its equipotent isomer, pregnanolone (PA), which allows for potent, positive, allosteric potentiation of the GABA_A_ receptors located in the post-synaptic membrane of pyramidal neurons of the frontal cortex and hippocampus, and pyramidal-like neurons of the basolateral amygdala (Agís-Balboa et al., 2006, 2007; Pinna et al., 2008).

These findings suggest that allopregnanolone functions downstream of PPAR–α to mediate its therapeutic effects (Figure 1), thus, we further hypothesize that part of the mechanism of action of PPAR–α includes an upregulation of the biosynthesis of neurosteroids (Raso et al., 2011), by upregulating the expression of crucial neurosteroidogenic enzymes. A recent study by Locci and Pinna (2017) further demonstrated the allopregnanolone-dependent effect of PPAR–α-activation. In this study, a single dose of a PPAR–α agonist, PEA or GW7647, normalized the levels of allopregnanolone in socially isolated mice, improved depressive-like and anxiolytic-like behavior, and facilitated impaired extinction of fear memory. The therapeutic-like effects of the PPAR–α agonists were however obstructed by genetic ablation of PPAR–α, antagonism of PPAR–α, and inhibition of neurosteroidogenic enzymes. This and previous studies further support a possible *PPAR–*α*-allopregnanolone biomarker axis* in PTSD, and a new therapeutic target for emotional disorders (discussed in Locci et al., 2018).

## Conclusion

Collectively, these observations provide a relevant case for the design of novel molecules. It suggests that activating PPAR–α may induce a downstream increase of neurosteroid biosynthesis, and that allopregnanolone, pregnanolone, and their analogs can be synthesized to mimic neurosteroid actions at GABA_A_ receptors. These can therefore provide important and novel steroid-based therapeutics for behavioral deficits in PTSD and other mood disorders. With overlapping symptoms spread across psychiatric disorders like PTSD, MDD and anxiety spectrum disorder, established methodical biomarkers will aid rapid differentiation, identification, prevention, and treatment of PTSD. Given the new relationship pointed out in this opinion article, the biochemical profile of PTSD may include a *PPAR–allopregnanolone biochemical axis* such that subpopulations of PTSD patients may display reduced allopregnanolone levels that can be increased by PPAR–α activation, only in allopregnanolone-deficient patients. Other components of the profile can also include changes in GABA_A_ receptor subunit expression (Locci and Pinna, 2017), decreased levels of endogenous fatty acid amides such as PEA and OEA (Hillard, 2018), or downregulated expression of PPAR–α. The mechanism by which stress induces changes in these neurochemical targets may be a potential *biomarker axis* relevant to diagnosis and as a novel approach to treat emotional and cognitive impairment in PTSD.

## Author contributions

KN wrote an initial draft of this opinion article. KN produced the graphics. GP revised the final version of the manuscript.

### Conflict of interest statement

The authors declare that the research was conducted in the absence of any commercial or financial relationships that could be construed as a potential conflict of interest.
